# COMBINED TECHNIQUES OF CAUDAL EPIDURAL BLOCK AND TRANSFORAMINAL NERVE ROOT BLOCK IN THE TREATMENT OF DEGENERATIVE DISEASES OF THE LUMBAR SPINE: A COST-EFFECTIVENESS ANALYSIS

**DOI:** 10.1590/1413-785220243205e276189

**Published:** 2024-10-28

**Authors:** Marília de Jesus Nogueira, Anderson Gomes Marin, Mariana Demétrio de Sousa Pontes, Carlos Fernando Pereira da Silva Herrero

**Affiliations:** 1. Universidade de São Paulo, Faculdade de Medicina, Departamento de Ortopedia e Anestesiologia, Ribeirao Preto, Sao Paulo, Brazil.; 2. Universidade de São Paulo, Faculdade de Medicina, Departamento de Ortopedia e Traumatologia, Ribeirao Preto, Sao Paulo, Brazil.; 3. Grupo São Lucas, Departamento de Ortopedia e Traumatologia Ribeirao Preto, Sao Paulo, Brazil.

**Keywords:** Cost-Effectiveness Evaluation, Local Anesthetics, Low Back Pain, Pain Measurement, Spinal Diseases, Visual Analog Scale, Avaliação de Custo-Efetividade, Anestésicos Locais, Dor Lombar, Medição da Dor, Doenças da Coluna Vertebral, Escala Analógica Visual

## Abstract

Objective: This study aims to assess cost-effectiveness of caudal epidural block with transforaminal nerve root block in the treatment of degenerative diseases of the lumbar spine. Methods: A total of 47 patients with lumbar sciatica symptoms were included. Low back pain and leg pain were assessed using the visual analogue scale (VAS), both in the pre-procedure and one week after. The cost-effectiveness and value required to improve each point on the VAS were estimated using addition, division, and rule of three calculations. Results: For low back pain, scores ranging from 2 to 10 were found before the procedure, with a mean of 7.5 ± 2.14 (95%CI: 6.8–8.1). A week after, these scores ranged from 0 to 10, with a mean of 3.1±2.8 (95%CI: 2.3–4.0; p < 0.0001). Regarding leg pain, scores ranging from 1 to 10 were noted before the procedure, with a mean of 6.8 ± 2.5 (95%CI: 6.1–7.4). A week after, these scores ranged from 0 to 9, with mean of 2.4 ± 2.5 (95%CI: 1.8–3.1; p < 0.0001). The cost of the materials used during the procedure was 214.72 BRL. Conclusion: Caudal epidural with transforaminal nerve root block were a cost-effective treatment modality for patients with degenerative diseases of the lumbar spine. ***Level of evidence III, Retrospective cohort study.***

## RESUMO

Objetivo: Testa a avaliação de custo-efetividade do bloqueio epidural sacral com bloqueio foraminal no tratamento de doenças degenerativas da coluna lombar. Métodos: Foram incluídos 47 pacientes com sintomas de ciática lombar. A avaliação da dor lombar e dor nas pernas foi quantificada pelo uso da escala visual analógica (VAS), antes do procedimento e uma semana depois. A custo-efetividade e o valor necessário para melhorar um ponto na VAS foram calculados usando adição, divisão e regra de três. Resultados: Para a dor lombar, foi observada uma variação de 2 a 10, com média de 7,5±2,14 (IC95%: 6,8-8,1) antes do procedimento; e uma variação de 0 a 10, com média de 3,1±2,8 (IC95%: 2,3-4,0) uma semana após (p < 0,0001). Para dor nas pernas, uma variação de 1 a 10 foi observada, com média de 6,8 ± 2,5 (IC95%: 6,1-7,4) antes da intervenção; e uma variação de 0 a 9, com média de 2,4 ± 2,5 (IC95%: 1,8-3,1) uma semana depois (p < 0,0001). O custo dos materiais utilizados durante o procedimento foi de 214,72 reais. Conclusão: O bloqueio epidural sacral e o bloqueio foraminal foram modalidades de tratamento com custo-efetividade para pacientes com doenças degenerativas da coluna lombar. ***Nível de evidência III, Estudo de coorte retrospectivo.***

## INTRODUCTION

 Spinal degenerative diseases can affect up to 65% of the world’s population annually and up to 84% of people at some moment in their lives. ^1,^ The most common symptoms are low back pain and sciatica. ^2^ Such symptomatology depends on which findings are present, such as facet joint arthrosis, spondylolysis, foraminal stenosis, disc herniation, spondylolisthesis, and degenerative disease of the intervertebral disc. ^2^ ^,^ ^3^

 Several authors have demonstrated an association between the symptoms resulting from these alterations and the reduction in the patient’s quality of life. ^5^ ^,^ ^6^ Thus, the treatment aims to improve the patient’s clinical conditions and quality of life. ^5^ Concerning therapeutic options, there is a diversity of treatments available, ranging from fixations with arthrodesis to non-surgical measures such as medication and physiotherapy. ^8^ It is well-defined that Each diagnosis require a particular treatment, which should be pointed out based on the patient’s symptoms and complementary exams. ^8^ ^,^ ^9^

 A treatment that has been increasingly used for symptoms resulting from degenerative spinal disease involves administering facet-joint and epidural injections, emphasizing the caudal epidural injection and transforaminal injection. ^7^ ^,^ ^11^ The terminology used to define epidural injections is already challenge among spine and pain specialists. ^14^ ^,^ ^18^ Terms such as facet denervation, percutaneous rhizotomy, paraspinous infiltration, nerve injection, percutaneous neurolysis, sympathetic infiltration, foraminal infiltration, facet injection, sacral injection, and epidural infiltration are some of the terms in the Brazilian Unified Supplementary Health Terminology (TUSS) table, thus recognized by the Brazilian National Supplementary Health Agency (ANS), which are used to designate a type of percutaneous intervention aimed at treating low back pain. ^16^ ^,^ ^20^ This difficulty results in heterogeneity in the analysis of results after infiltrations when we look for evidence in the literature that supports its prognosis. ^12^ ^,^ ^15^ ^,^ ^17^

 Currently, there are several specific materials available that can be used to carry out such interventions, ranging from spinal anesthesia needles, available at any health unit, to cooled radiofrequency cannulas, which are expensive, with varying costs, and less available on the market, impacting the necessary expense to treat a patient. ^19^ ^,^ ^20^

 Despite being considered a less invasive pain management technique, epidural injection is not exempt from complications. ^12^ Pain worsening, neuropraxia, meningeal lesions, abscess formation, and even paraplegia are described as complications of epidural injections and must be considered when indicating a specific procedure. ^17^ ^,^ ^20^

Thus, this study aims to investigate the cost-effectiveness and complications of combining procedures, transforaminal and caudal injections, in treating patients with degenerative lumbar spine diseases associated with low back pain and sciatica.

## METHODS

### Ethical aspects

This study is an observational, retrospective cohort study with a quantitative and qualitative approach and was approved by the Research Ethics Committee of the institution where the study was carried out (CAAE:48835721.8.0000.8098), fulfilling the prerogatives of Resolution no. 466/2012 of the Brazilian National Health Council regarding the parameters of research with human beings.

### Sample characteristics

In total, 47 patients diagnosed with degenerative lumbar spine disease and symptoms of low back pain associated with sciatica were included in the study. All data were obtained from the medical records of patients treated at the same private clinic by the same physician with more than 10 years of specialization in spine surgery and pain management.

Patients of both sexes, aged over 18 years, with low back pain and/or pain radiating to the leg that was refractory to conservative treatment with analgesic medication and physiotherapy for more than four weeks were included. All patients underwent a combination of caudal epidural and transforaminal injections. Exclusion criteria included patients under 18 years, those diagnosed with a tumor disorder, infection, or spinal trauma, and those with a history of previous lumbar spine surgery. Complementary imaging, such as radiography and magnetic resonance imaging (MRI) of the spine, was used in all cases to confirm the diagnosis.

### Analyzed variables

The information collected from the patient’s medical record included age, sex, diagnosis, occurrence of complications resulting from the procedure, initial pain intensity (iVAS), and pain intensity one week after the procedure (fVAS). Pain levels were assessed using Visual Analog Scale (VAS). VAS scores were recorded for both low back pain and leg pain.

Moreover, data on the total time and cost of the procedure were collected from hospital records. The procedure cost was estimated from the sum of all devices, materials, and medications used to treat each patient. Information was also obtained from the patient’s medical records.

The cost-effectiveness (CE) was estimated by dividing the procedure cost by the mean difference between the iVAS and the fVAS, as shown below:

Cost-effectiveness (CE) = Total cost (TC) / (iVAS − fVAS)

The cost required to improve by one point on the VAS was estimated using the rule of three, as presented below:

Total procedure cost (TC) ----------------- (iVAS − fVAS)

Cost of X ----------------------------------------------- 1 point on VAS

The cost of X is the cost required to improve one point on the visual analog pain scale (VAS).

### Procedures

The procedures were performed at the same hospital by the same spine surgeon, using the same mobile C-arm device (Model: GE OCE Fluorostar Compact; Manufacturer: GE OCE MEDICAL SYSTEMS GMBH; Serial number: FCDxxA18120685; Date of manufacture: December/2018; Made in Germany). All patients were administered transforaminal injection (1.5 to 2 ml of solution per foramen) using a solution containing 1 ml of 2% lidocaine 20 mg/ml without vasoconstrictor (xylestesin) and 1 ml of triamcinolone hexacetonin 20 mg/ml (Triancil). For caudal epidural injection, a solution containing one ampoule of betamethasone diproptonate 5 mg + disodium phosphate 2 mg (Eurofarma) and one ampoule of lidocaine 2% 20 mg/ml without vasoconstrictor (xylestesin).

Before performing the procedure, the attending physician met the patient in the waiting room and asked about the intensity of pain using the VAS, recorded as the iVAS. Then, the patient was taken to the operating room, positioned in the horizontal prone position, and submitted to anesthetic sedation. Thus, asepsis was performed, and appropriate sterile fields were placed. With the positioning and use of the mobile C-arm in the anteroposterior view, the desired lumbar level was identified and, with the oblique view, the needle was inserted into the foramen, and after confirming its proper positioning, the analgesic solution was injected. These steps were repeated according to the number of foramina affected.

Afterwards, the mobile C-arm was positioned on the lateral view at the level of the sacrococcygeal region, and the sacral hiatus was palpated. The needle was inserted into the sacral hiatus, and its proper positioning was confirmed by administering radiopaque contrast 300 mg/ml (Omnipaque 50 ml), and the analgesic solution was subsequently injected.

The patient was then directed to the post-anesthesia recovery room and discharged according to the criteria of the anesthesia team. A week after the procedure, the attending physician in charge scheduled a new appointment with the patient and recorded the fVAS.

### Statistical analysis

Data tabulation was performed using the Microsoft Excel® 2016 software. The data obtained were statistically analyzed using the Stata ® 2018 software, and the mean and standard deviations were used. For inferential analysis, the Student’s t-test for paired samples was used.

## RESULTS

A total of 47 patients with degenerative spine diseases associated with low back pain and sciatica met the inclusion criteria and were selected for the study. In total, 17 males and 30 females were included, aged 24 to 86 years, with a mean age of 56.4±17.23 years. The age of female patients ranged from 26 to 84 years, with a mean of 60.13 years, whereas male patients’ age ranged from 24 to 86 years, with a mean of 49.17 years. Among the diagnoses of the evaluated patients, we found facet degeneration, intervertebral disc degeneration, spinal canal stenosis, disc herniation, and patients with grade 1 spondylolisthesis. Regarding the complications, no occurrences were identified.

 Low back pain assessment using the VAS found values ranging from 2 to 10, with a mean of 7.5 ± 2.14 (95%CI 6.8 – 8.1) before the procedure (iVAS). A week after (fVAS), it ranged from 0 to 10, with a mean of 3.1 ± 2.8 (95%CI 2.3 – 4.0; p < 0.0001) . ( Table 1 ) 

 Meanwhile, leg pain assessment using the VAS found values ranging from 1 to 10, with a mean of 6.8 ± 2.5 (95%CI 6.1 – 7.4) before the procedure (iVAS). A week after (fVAS), it ranged from 0 to 9, with a mean of 2.4 ± 2.5 (95%CI 1.8 – 3.1; p<0.0001). ( Table 1 ) 

**Figure 1.**
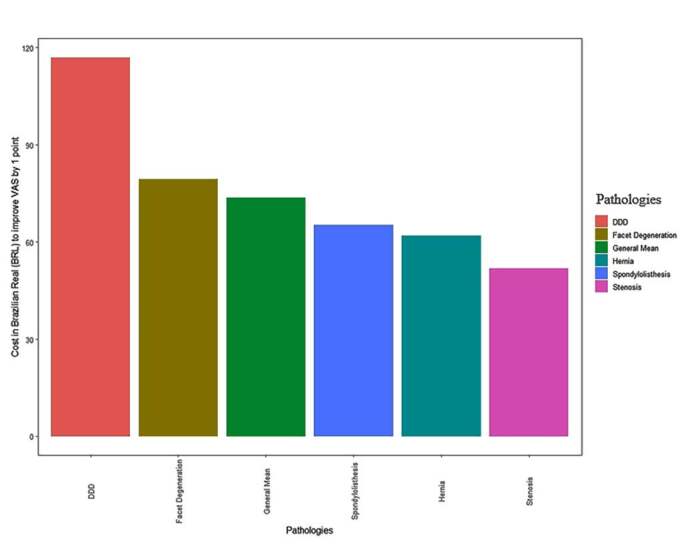
Cost-effectiveness for improving lumbar spine pain after epidural injection. Legend: DDD = degenerated disc disease.

**Figure 2.**
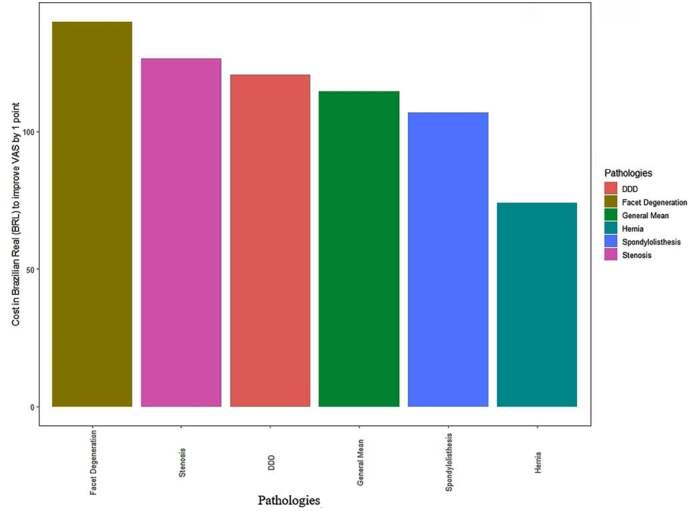
Cost-effectiveness for improving pain in lower limbs after epidural block. Legend: DDD = degenerated disc disease.

 The total cost of the procedure was 214.75 BRL, remaining the same for all patients included in the study ( Table 2 ). The cost-effectiveness (CE) for improving one point on the VAS for low back pain was 73.62 BRL ( Table 3 ) ( Figure 1 ), whereas that for leg pain was 114.51 BRL ( Table 4 ) ( Figure 2 ). 

**Table 1.** Assessment of lumbar pain (n = 45) and sciatica (n = 56) using the visual analog scale in patients undergoing epidural infiltration.

**Pain**	**Preoperative**	**Postoperative**	**p**
Lumbar Spine	7.5 ± 2.14 (IC95% 6.8 – 8.1)	3.1 ± 2.8 (IC95% 2.3 – 4.0)	< 0.0001
Lower limbs	6.8 ± 2.5 (IC95% 6.1 – 7.4)	2.4 ± 2.5 (IC95% 1.8 – 3.1)	< 0.0001

**Table 2.** Materials used to perform caudal epidural and transforaminal injection with respective amounts and values in reais.

**The material used**	**Quantity**	**Unit value (BRL)**	**Total value (BRL)**
Alfenthalin 0.5 mg/ml	1 ampoule	14.99	14.99
Midazolam 1 mg/ml	1 ampoule	5.27	5.27
Ringer lactate 500 ml	1 bag	2.14	2.14
Abocath 24G (0.7×119 mm)	1 unit	2.53	2.53
Nasal oxygen catheter	1 unit	0.91	0.91
Microdropper equipment	1 unit	3.82	3.82
Tegaderm peripheral cateter	1 unit	5.45	5.45
Needle 25 mm × 8 mm	1 unit	0.1	0.1
Spinal needle	3 units	13.56	40.68
10 ml syringe with thread	2 units	0.5	1
5 ml syringe with thread	1 unit	0.33	0.33
5 ml syringe without thread	1 unit	0.33	0.33
Betamethasone dipropionate + disodium phosphate 5 mg + 2 mg	1 unit	3.25	3.25
Non- ionic iodine contrast 300 mgi/ml	1 unit	34.68	34.68
Lidocaine 2%	1 unit	6.11	6.11
Triamcinolone 20 mg/ml	1 unit	81.2	81.2
Sterile operation field	1 unit	7.06	7.06
Chlorhexidine 0.5% alcoholic solution 100 m	1 unit	1.87	1.87
Sterile gauze	2 units	0.7	1.4
Surgical glove	1 unit	1.63	1.63
	-	-	214.75

mg: milligrams; ml: milliliter; mm: millimeters; % = percentage; BRL: Brazilian reais.

**Table 3.** Cost-effectiveness for improving lumbar spine pain after epidural injection.

Number of patients	**Pain improvement**	**Cost of X**	**Disease**
45	4.33	73.63	Overall Average
4	4.50	79.36	Facet Degeneration
11	2.09	116.94	Discopathy
16	5.25	51.81	Stenosis
10	4.90	61.94	Hernia
4	5.25	65.32	Spondylolisthesis

Cost of X: cost needed to improve one point on the visual analog scale.

**Table 4.** Cost-effectiveness for improving pain in lower limbs after epidural block.

**Number of patients**	**Pain improvement**	**Cost of X**	**Disease**
52	2.60	114.52	
3	1.67	139.76	Facet Degeneration
10	1.90	120.56	Discopathy
25	2.28	126.48	Stenosis
10	4.60	74.12	Hernia
4	2.00	106.69	Spondylolisthesis

Cost of X: cost needed to improve one point on the visual analog scale.

## DISCUSSION

The combination of caudal epidural and transforaminal injections proved to be, in this study, a cost-effective treatment modality with a low complication rate for patients with degenerative diseases of the lumbar spine.

 Similar studies have demonstrated that epidural injections do not change the evolutionary process of the disease, ^11^ ^,^ ^14^ ^,^ ^17^ but rather offer immediate relief of the patient’s pain, allowing earlier rehabilitation. ^10^ ^,^ ^20^ Moreover, it can be considered a minimally invasive and low-cost procedure compared to invasive surgical procedures, with a low risk of complications. ^10^ Among the injections, the lumbar transforaminal and the caudal epidural injection have been used in treating low back pain and sciatica caused by degenerative diseases of the spine. ^18^ ^,^ ^20^

 Numerous studies, including contemporary publications by Manchikant, Lee, and Chou, have highlighted that epidural injection provides moderate short-term pain improvement. ^8^ ^,^ ^16^ However, epidural injections are not superior in the long term compared to conservative treatment alone. ^19^ Similar results were found in studies by Pennington, in which, after performing the injection, improvements were noted in the patient’s quality of life during the first three months, which was not maintained during the six months of follow-up. ^20^ Our results align with these findings, as we observed a considerable improvement in pain one week after the procedure, both for low back pain and sciatica. Despite this, long-term pain improvement was not considered in this study since, with short-term improvement, patients could more easily perform rehabilitation and strengthening exercises in physiotherapy sessions. 

A variable that we studied but found no comparable research, was the cost necessary to improve the patient’s quality of life, represented by the VAS. We demonstrated that a small cost would be required to improve one point on the VAS for low back and leg pain, respectively 73.62 BRL and 114.51 BRL.

We highlight that our study shows limitations. Firstly, we underscore that this is an observational analysis that used medical records of patients with symptoms resulting from the degenerative spinal disease who had unsuccessfully submitted to treatment with physiotherapy and oral medications. Secondly, we did not apply any quality of life questionnaires, which could provide more information about the impact of the disease and resulting symptoms on patients’ daily routines. Finally, the diversity of diagnoses and possible sources of similar symptoms hinder generalization of results.

However, our study also presents strengths, as we used a standardized treatment for low back pain and sciatica for degenerative diseases of the lumbar spine, using the same devices and mobile C-arm for all injections. Moreover, the same spine surgeon performed all procedures in the same hospital, and data were collected by the same assistant.

## CONCLUSION

Our results showed that combining caudal epidural and transforaminal injection procedures is a cost-effective treatment modality for patients with degenerative lumbar spine diseases associated with symptoms of low back pain and sciatica for a short period. In addition, the procedure proved to be safe, with a low complication rate.
